# Editorial: Potential therapeutic approaches of female hormones in the brain

**DOI:** 10.3389/fphar.2025.1617394

**Published:** 2025-05-27

**Authors:** Pelin Kelicen Ugur, Mariagiovanna Cantone

**Affiliations:** ^1^ Department of Pharmacology, Faculty of Pharmacy, Hacettepe University, Ankara, Türkiye; ^2^ Neurology Unit, Policlinico University Hospital “G. Rodolico-San Marco”, Catania, Italy

**Keywords:** estrogen, neurodegeneration, hormone/reproduction/sexual, gender, women health and psychology

## Introduction

Sex and gender differences play a crucial role in shaping health outcomes across a range of medical conditions. From the use of oral anticoagulants in atrial fibrillation to the impact of hormone replacement therapy on cognitive function and mental health, biological and sociocultural factors must be considered to optimize treatment strategies. This Research Topic explored the intersection of sex differences in pharmacological interventions, with a particular focus on anticoagulant therapy, opioid response, neurodegenerative diseases, gut health and hormone-based treatments. By synthesizing current research on these topics, we aim to highlight the need for personalized medicine approaches that take into account sex- and gender-specific variations in drug efficacy and safety. Addressing these disparities will not only enhance clinical decision-making but also contribute to more equitable healthcare practices that improve patient outcomes.

This Research Topic aimed to bring together groundbreaking studies to deepen our understanding of the molecular, genetic, and clinical aspects of sex and gender differences in brain health and related diseases. Between February 2023 and February 2025, we received numerous high-quality submissions. After a rigorous peer-review process, five outstanding articles were selected for publication. Collectively, these contributions advanced our knowledge of female hormones in the brain and suggested potential avenues for future research and therapeutic strategies.

Giner-Soriano et al. described the dose adequacy in patients receiving direct oral anticoagulants, finding a high frequency of underdosing that was significantly higher in women in comparison with men. They highlighted the need for improved sex- and gender-conscious prescribing practices to optimize treatment efficacy and safety. The observed disparities in anticoagulant use underscore the importance of personalized medicine approaches that consider both biological and sociocultural factors in the management of atrial fibrillation. Further research is required to evaluate the long-term clinical outcomes of these differences and to promote equitable healthcare strategies in anticoagulant therapy. Addressing these sex- and gender-related gaps will enhance stroke prevention strategies and ensure optimal patient care in diverse populations.

Hormone Replacement Therapy (HRT) has been shown to be effective in managing cachexia and improving quality of life in cancer patients, although mild side effects can occur. Concerns about the risks and benefits of estrogen therapy—particularly its potential to induce tumor recurrence or metastasis in breast cancer, endometrial carcinoma, ovarian cancer, and gastric cancer—limit its use, despite its protective effects against colorectal and liver cancer. Therefore, Zhu et al. reviewed the use of progesterone as a potential treatment for patients suffering from pain, depression, and cachexia. While treatments with rapid effects are more likely to gain approval, long-term studies are essential to understand the broader impact of hormone therapies. Overall, while careful consideration of safety and benefit is crucial, the potential of hormone therapies for brain health and symptom management should not be overlooked.

The study by Conley et al. has significant implications for understanding the role of HRT in the cognitive health of postmenopausal women, particularly those at risk for Alzheimer’s disease (AD). The authors focused on women at a higher risk of developing AD, and the decline in estrogens post-menopause is thought to be a factor that increases this risk. They found significant impairments on some tasks when participants took estradiol (E2) together with micronized progesterone (mPRO) compared to E2 alone. Their findings show that when mPRO was administered alongside E2, it impaired effortful cognitive performance under conditions of cholinergic blockade. These results are important for assessing the impact of combined postmenopausal hormone treatment on cognitive performance, which is dependent on cholinergic function after menopause. This highlights the need for further research on the effects of different progestins on cholinergic-mediated cognitive performance and their relationship to long-term cognitive decline.

Strong evidence suggests that both sex hormones and opioids influence the composition and function of the gut microbiome and opioid drugs have long been known to induce different responses in men compared to women. Therefore, the molecular mechanisms underlying these effects and how sex differences in opioid responses are shaped by both central nervous system mechanisms and peripheral factors, including interactions between the gut microbiome and hormones should be fully characterized. Han et al. emphasized the need for future research to better understand the interplay between sex and the microbiome, which is critical for improving clinical applications and developing more personalized and effective opioid treatment strategies.

HRT also offers promising options for managing menopausal depression by addressing hormonal imbalances that contribute to mood fluctuations, in addition to supporting reproductive health or protecting against neurodegenerative diseases such as AD. Therefore, Mu et al., focused on the potential of gonadal hormone therapies to treat depression in women by targeting hormonal imbalances that affect mood. However, given the variability in individual responses, personalized treatment plans that take into account hormone type, dosage, and administration method are essential. Emerging hormone treatments offer promising solutions, but further research—especially large-scale randomized controlled trials—is needed to evaluate these therapies. Addressing these research gaps will help optimize treatment strategies and improve the quality of life for women affected by depression.

## Conclusion

Understanding the influence of sex and gender on pharmacological responses is essential for developing more effective and equitable treatment strategies. The findings reviewed in this topic emphasize the need for continued research into sex-specific differences in medication efficacy, particularly in anticoagulant use, opioid response, neurodegenerative diseases, mood disorders, gut health and hormone-based therapies. Personalized treatment approaches that consider biological and sociocultural factors will be instrumental in optimizing patient care. Moving forward, large-scale studies and clinical trials should be prioritized to bridge existing knowledge gaps and refine therapeutic strategies that enhance health outcomes for diverse populations. By incorporating sex- and gender-conscious medical practices, we can pave the way for more precise and inclusive healthcare solutions.

## Future perspectives

The research presented in this Research Topic underscores the multifaceted nature of hormone-related diseases, particularly in women, and the need for an interdisciplinary approach to unravel their complexities. From neuroinflammation to genetics to microbiome influences, these studies provide valuable insights that pave the way for future advancements in the field.

As this Research Topic concludes, we extend our sincere gratitude to all of the contributing authors, reviewers, and researchers who participated in this initiative. We hope that the findings presented herein will inspire further investigation and contribute to the development of innovative therapeutic and diagnostic strategies for hormone-related diseases in women.

To access the full Research Topic of articles, please visit: Potential Therapeutic Approaches of Female Hormones in the Brain.

